# Magnetic localization and control of helical robots for clearing superficial blood clots

**DOI:** 10.1063/1.5090872

**Published:** 2019-05-20

**Authors:** Islam S. M. Khalil, Alaa Adel, Dalia Mahdy, Mina M. Micheal, Mohanad Mansour, Nabila Hamdi, Sarthak Misra

**Affiliations:** 1 Department of Biomechanical Engineering, University of Twente, Enschede 7500 AE, The Netherlands; 2 Department of Mechatronics Engineering, The German University in Cairo, New Cairo 11835, Egypt; 3 Department of Pharmacology and Toxicology, The German University in Cairo, New Cairo 11835, Egypt; 4 Department of Biomedical Engineering, University of Groningen and University Medical Centre Groningen, Groningen 9713 AV, The Netherlands

## Abstract

This work presents an approach for the localization and control of helical robots during removal of superficial blood clots inside *in vitro* and *ex vivo* models. The position of the helical robot is estimated using an array of Hall-effect sensors and precalculated magnetic field map of two synchronized rotating dipole fields. The estimated position is used to implement closed-loop motion control of the helical robot using the rotating dipole fields. We validate the localization accuracy by visual feedback and feature tracking inside the *in vitro* model. The experimental results show that the magnetic localization of a helical robot with diameter of 1 mm can achieve a mean absolute position error of 2.35 ± 0.4 mm (*n *=* *20). The simultaneous localization and motion control of the helical robot enables propulsion toward a blood clot and clearing at an average removal rate of 0.67 ± 0.47 mm^3^/min. This method is used to localize the helical robot inside a rabbit aorta (*ex vivo* model), and the localization accuracy is validated using ultrasound feedback with a mean absolute position error of 2.6 mm.

Magnetic microrobots hold promise in targeted drug delivery by enabling interventions with minimal incisions and access to deep-seated-regions of the human body.^1–3^ The power (batteries or power supplies) and mechatronic systems (controllers and sensors) required to control these microrobots are separated and embedded into an actuation system,^4^ thereby significantly simplifying the microrobot design into a wire formed into a helix^5–9^ or an elastic tail.^10,11^ The integration of imaging modalities to the actuation systems allows us to translate microrobots into *in vitro* preliminary experiments and *in vivo* trials.^12–17^ With magnetic actuation, even more so than with other actuation techniques,^18,19^ we can use the actuating magnetic field for propulsion and localization.^20,21^ Therefore, it may be possible to localize and control the microrobot even without a traditional imaging modality. For instance, Popek *et al.* have demonstrated simultaneous localization and propulsion of a magnetic capsule in a lumen using a single rotating dipole field.^22^ They have designed an extended Kalman filter to estimate the capsule's six-degrees-of-freedom pose as it is synchronized with the applied magnetic dipole field. This level of simultaneous localization and control has been achieved by embedding six Hall-effect sensors into a relatively large capsule with 42 mm in length. Di Natali *et al.* have also presented a real-time pose detection that combines multiple sensors with a precalculated magnetic field map.^23^ Yim and Sitti have utilized magnetically actuated shape deformation and recovery to localize a magnetically actuated soft capsule endoscope between rolling locomotion cycles.^24^

All prior magnetic-based propulsion and localization methods have utilized a relatively large capsule to contain a permanent magnet and magnetic field sensors. To implement this approach on microrobots, it is not viable to use on-board magnetic field sensors and maintain a simple design that can be scaled down to enable access to difficult-to-reach locations in the body. Son *et al.* have utilized a five-degrees-of-freedom localization method for a meso-scale (6.4 × 6.4× 12.8 mm^3^) magnetic robot.^25^ They have introduced a two-dimensional array of Hall-effect sensors to measure the robot's magnetic fields using the modeled field of the actuating omnidirectional electromagnet. In order to implement this method on microrobots, with relatively low magnetic strength, the workspace will be significantly limited.

In this work, we localize helical robots with a diameter of 1 mm using an array of Hall-effect sensors and the precalculated magnetic field map of a permanent magnet-based robotic system.^26^ The potential application of this localization and estimation-based motion control method is the mechanical removal of blood clots in the superficial veins of the leg; the long and short saphenous veins (Fig. 1); a condition called superficial vein thrombosis (SVT). SVT in lower limbs is a common condition characterized by the formation of a blood clot in the superficial veins in the subcutaneous tissue (the innermost layer of skin). Although this condition has been previously reported to be benign, clinical studies have shown that SVT in the long saphenous vein of the leg could lead to major complications such as propagation into the deep veins with a risk of subsequent pulmonary embolism.^27^ The standard conservative therapy does not prevent the extension of the thrombus;^28^ thus the mechanical removal of SVT could be a promising minimally invasive therapeutic approach. The depth of these veins ranges between 1.5 mm and 31.6 mm,^29,30^ and the helical robot can be administrated into the corresponding vein using a flexible surgical instrument or a catheter. An advantage of helical robots over flexible surgical devices is their ability to access locations of the body that are inaccessible to tethered devices. Therefore, several research groups have utilized magnetically powered micro- and nanomotors to achieve enhanced thrombolysis.^31–33^ Closed-loop motion control of the helical robot is achieved based on its estimated position toward blood clots inside *in vitro* and *ex vivo* models. In the *in vitro* experiment, results of the localization are validated using visual feedback and feature tracking,^34^ whereas ultrasound feedback is used to validate the localization of the *ex vivo* trials.

**FIG. 1.**
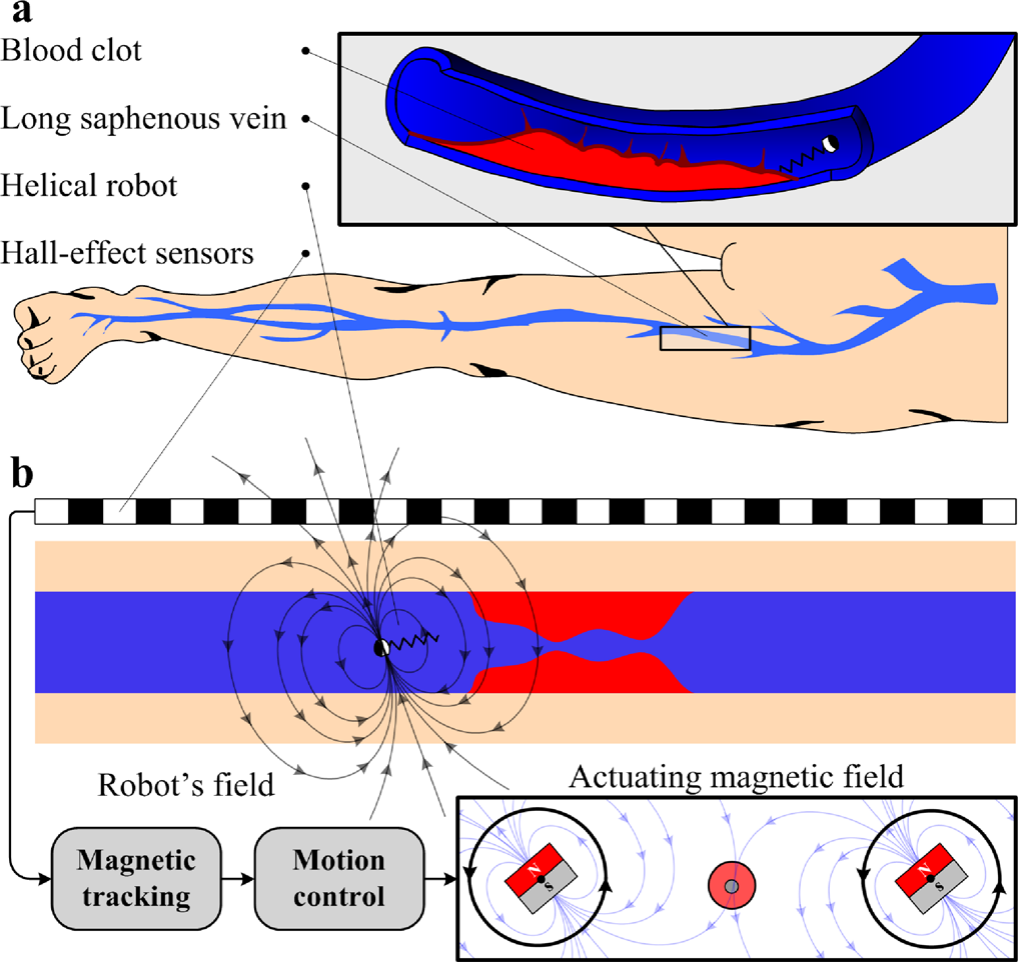
Localization and closed-loop motion control of a helical robot with a diameter of 1 mm are utilized in clearing blood clots *in vitro*. (a) The long and short saphenous veins (superficial veins) of the leg are commonly affected by thrombosis. (b) An array of Hall-effect sensors can be mounted along a superficial vein and used to localize a helical robot. The helical robot is actuated using two rotating dipole fields and closed-loop control is achieved based on its estimated position.

The remainder of this paper is organized as follows: Sec. I provides insights into the modeling of the helical robot, the magnetic localization and position estimation, and descriptions of the magnetic localization and actuation systems. Magnetic localization experiments and closed-loop motion control of the helical robot are provided in Sec. II and validated using visual and ultrasound feedback for the *in vitro* and *ex vivo* models of the blood clot, respectively. Section III provides discussions pertaining to the limitations and potential applications of the magnetic localization of helical robots. Finally, Sec. IV concludes and provides directions for future work.

## I. LOCALIZATION OF THE HELICAL ROBOT

The helical robot is actuated using two rotating dipole fields and localized while it is swimming inside a catheter segment (*in vitro* model) or a rabbit aorta (*ex vivo* model) via an array of Hall-effect sensors.

### A. System description

**FIG. 2.**
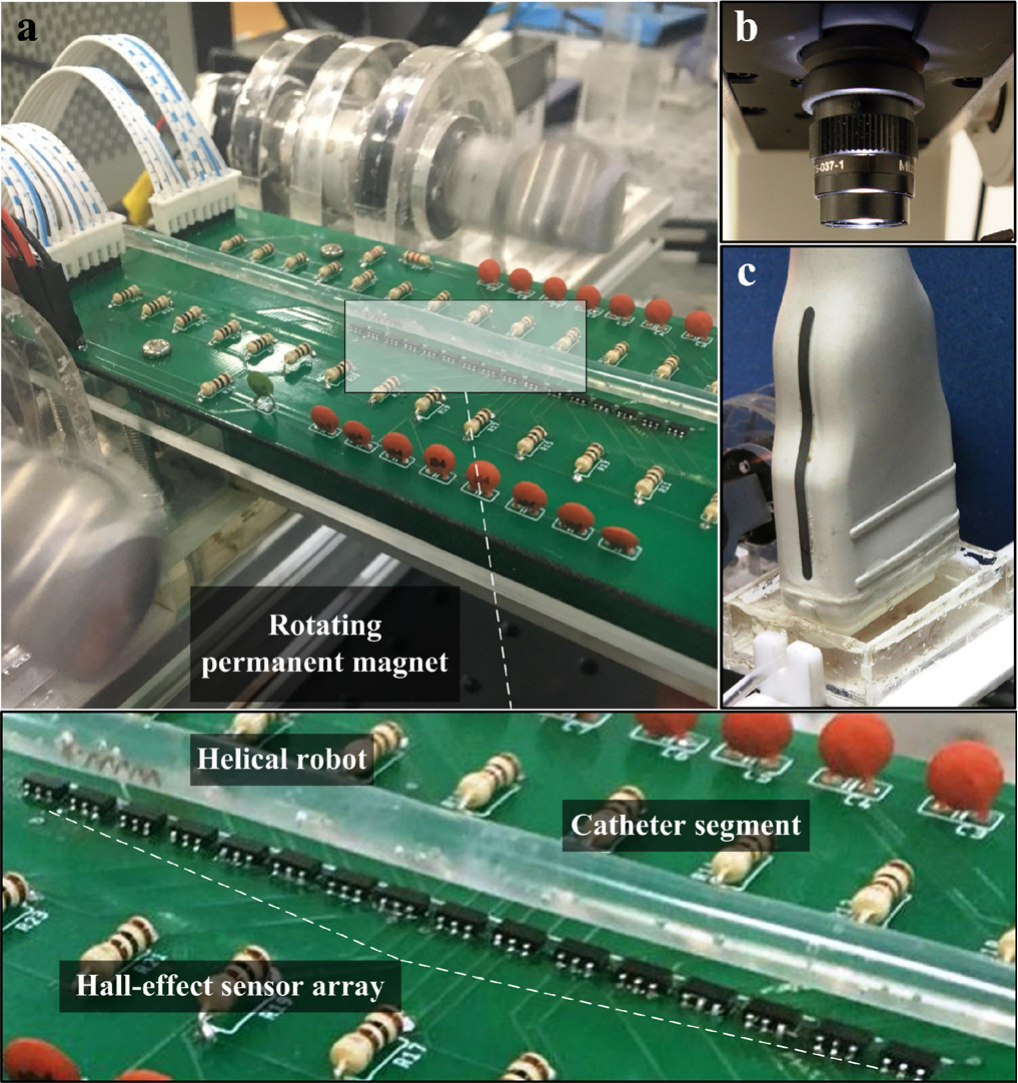
A permanent magnet-based robotic system enables a helical robot to swim using rotating magnetic fields. (a) A catheter segment is aligned with an array of Hall-effect sensors (3D magnetic sensor TLV493D-A1B6, Infineon Technologies AG, Munich, Germany). Position of the helical robot inside the catheter segment is estimated using measurements of these sensors and the precalculated magnetic field map of the rotating permanent magnets. (b) Position of the helical robot is measured with a high-speed camera (avA100-120kc, Basler Area Scan Camera, Basler AG, Ahrensburg, Germany) to validate the magnetic tracking. (c) An ultrasound transducer (LA523 linear array ultrasound transducer, Esaote, Italy) localizes the helical robot inside the *ex vivo* model.

Our system (Fig. 2) comprises *in vitro* and *ex vivo* models of the blood vessel, a permanent magnet-based robotic system, and an array of Hall-effect sensors. The *in vitro* model consists of a polyvinyl chloride catheter segment with an inner-diameter of 4 mm filled with phosphate buffered saline (PBS), with a viscosity of 0.8882 cP. Blood clots (1-h-old) are inserted into the catheter segment in each trial and PBS is injected at a flow rate of 10 ml/h, using a dual syringe pump (Genie Plus, GT-4201D-12, Kent Scientific, Connecticut, USA). This flow rate is devised based on the administration and infusion rates for adult patients.^35^ Motion of the helical robot is tracked with a high-speed camera (avA100–120kc, Basler Area Scan Camera, Basler AG, Ahrensburg, Germany) in the *in vitro* trials.

In the case of the *ex vivo* model, a segment from the aorta is isolated from a rabbit and connected to the catheter, and the camera is replaced by an ultrasound transducer (LA523 linear array ultrasound transducer, Esaote, Italy) for tracking using an ultrasound system (MyLab^TM^ X5 Ultrasound Imaging System, Esaote, Italy). A helical robot (diameter of 1 mm) is also inserted and allowed to swim toward the clot against the flowing streams of the PBS. The robot consists of a helical body and a permanent magnet with magnetization vector perpendicular to the helix axis. The robots are fabricated using a copper spring with a length, diameter, and pitch of 4 mm, 0.9 mm, and 0.85 mm, respectively. This spring is rigidly attached to a cylindrical NdFeB magnet. The relation between the geometric shape of the robot and the swimming speed has been characterized experimentally by Zhang *et al.* and Tottori *et al.*^6,7^ The robot is actuated using two synchronized rotating dipole fields. These fields are generated using permanent NdFeB magnets with a diameter of 20 mm and length of 20 mm, and axial magnetization. The distance between the axes of the rotating permanent magnets is 150 mm. Each magnet is attached to a DC motor (2322 980, Maxon Motor, Sachseln, Switzerland). The angular positions of these motors are synchronized to increase the magnetic field and mitigate the magnetic force along the lateral direction of the robot. A linear array of 16 Hall-effect sensors (3D magnetic sensor TLV493D-A1B6, Infineon Technologies AG, Munich, Germany) is fixed below the catheter segment, at a maximum height of 5 mm. The distance between the adjacent sensors is 1 mm, and their sensitivity is 0.1 mT within a range of ±130 mT (Table I).

**TABLE I.** Specification of the actuation and localization system of the helical robot. **M**_1,2_ and *ω* are the magnetization and rotational frequency of the permanent magnets. **m**, *D*, and *L* are the magnetization, diameter, and length of the helical robot, respectively. *f*, TIS, and MI are the frequency of the ultrasound waves, thermal index, and mechanical index, respectively. *μ*, *ρ*, and *v*_0_ are the viscosity and density of the medium and the initial volume of the blood clot, respectively.

Subsystem	Property	Value	Property	Value
Hall-effect	Sensitivity (mT)	0.1	Noise (mT)	0.1
sensor	Range (mT)	±130	Range (mm)	5
Rotating	Distance (mm)	150	*ω* (Hz)	5
dipoles	**M**_1,2_ (A m^2^)	6.087	Field (mT)	20
Helical	Type	NdFeB	*D* (mm)	1
robot	**m** (A m^2^)	1.7 × 10^–4^	*L* (mm)	5
Ultrasound	*f* (MHz)	12	TIS	0.1
system	MI	0.9	Gain	49
*In vitro* and	*μ* (cP)	0.8882	*ρ* (kg m^–3^)	995
*ex vivo*	*v*_0_ (mm^3^)	94.24	Flow (ml/h)	10

### B. Magnetic localization of the helical robot

**FIG. 3.**
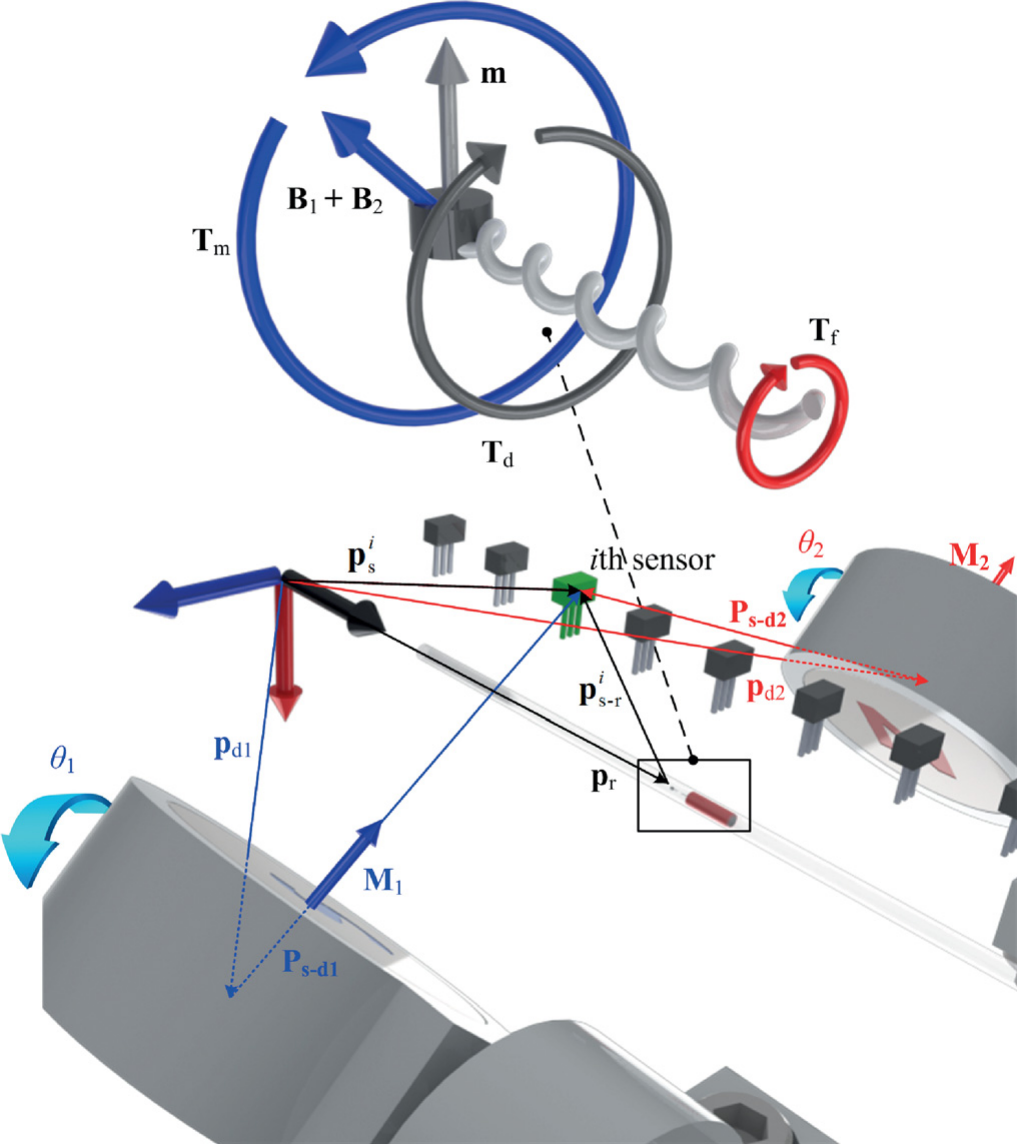
Dipole models of the helical robot (shown magnified) and the two rotating permanent magnets are used to localize the helical robot. The helical robot (with magnetization **m**) is contained inside a catheter segment between the two rotating permanent magnets with magnetization **M**_1_ and **M**_2_. $p_{s}^{i}$ is the position vector to the *i*th sensor from a reference frame and $p_{s-r}^{i}$ is position vector to the *i*th sensor from the robot's frame of reference. **p**_d1_ and **p**_d2_ are position vectors to the first and second rotating permanent magnets from the reference frame, respectively. The axis of rotation of the dipole fields is parallel to the axis of the helical robot and the catheter segment. **B**_1_ and **B**_2_ are the fields of the permanent magnets and exert magnetic torque **T**_m_ to overcome the drag torque (**T**_d_) and fretting torque (**T**_f_), with the fluid and clot, respectively.

The helical robot consists of a cylindrical permanent magnet with magnetization vector (**m**) perpendicular to its helix axis. A magnetic torque is applied using two dipole fields **B**_1_ and **B**_2_, as shown in Fig. 3. These fields are generated using two rotating permanent magnets with dipole moment **M**_1_ and **M**_2_. Therefore, the *i*th Hall-effect sensor is subject to the following magnetic fields:

$$B_{s}^{i}=B_{r}+B_{d1}+B_{d2},$$ (1)

where $B_{s}^{i}$ is the magnetic field at the *i*th sensor due to the robot and the two dipole fields, and **B**_r_ is the magnetic field of the helical robot. Further, **B**_d1_ and **B**_d2_ are the fields of the first and second dipole fields at the *i*th sensor. The magnetic field of the helical robot is given by^22^

$$B_{r}=\frac{\mu_{0}\left|m\right|}{4\pi }\left(\frac{3\left(\hat{m}\cdot p_{s-r}^{i}\right)p_{s-r}^{i}-{\left|p_{s-r}^{i}\right|}^{2}\hat{m}}{{\left|p_{s-r}^{i}\right|}^{5}}\right),$$ (2)

where *μ*_0_ is the magnetic permeability of free space and $\hat{m}$ is the unit vector of the magnetization vector of the helical robot. Further, $p_{s-r}^{i}$ is the position vector to the *i*th sensor from the helical robot's frame of reference. In (1), the magnetic field of the first and second rotating permanent magnets is calculated using^22^

$$B_{dj}=\frac{\mu_{0}\left|M_{j}\right|}{4\pi }\left(\frac{3\left({\hat{M}}_{j}\cdot p_{dj}\right)p_{dj}-{\left|p_{dj}\right|}^{2}{\hat{M}}_{j}}{{\left|p_{dj}\right|}^{5}}\right),$$ (3)

where **M**_*j*_, for *j *=* *1, 2, is the magnetic moment of the *j*th permanent magnets and ${\hat{M}}_{j}$ is its unit vector. Further, **p**_d__*j*_ is position vector to the *i*th sensor from the *j*th permanent magnet.

**FIG. 4.**
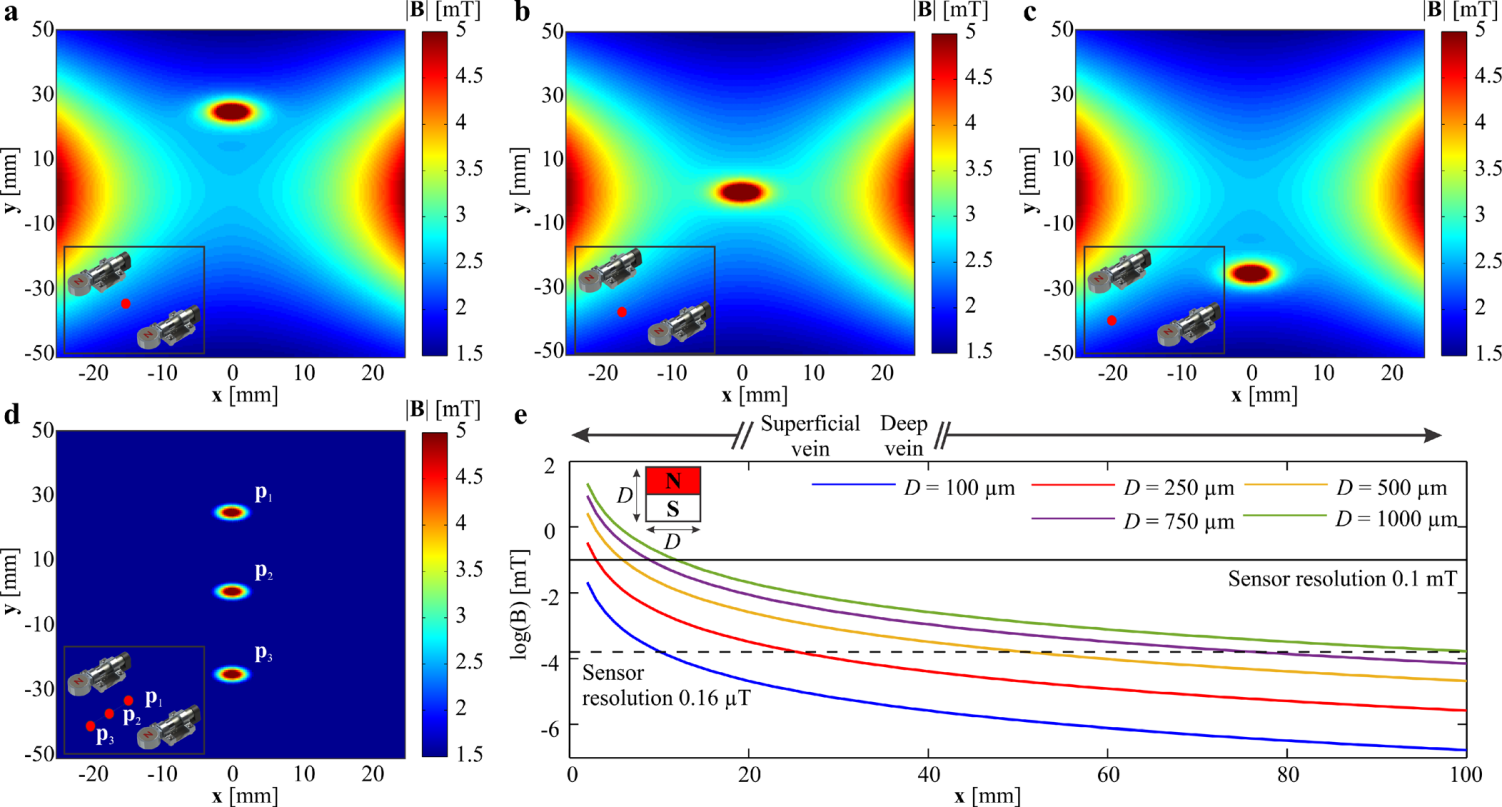
The precalculated magnetic field map of the two rotating permanent magnets (zero angular position) is superimposed to the helical robot's magnetic field and calculated at the plane of the Hall-effect sensor (**x**, **y**, 3) mm. The distance between the rotating permanent magnets is 15 cm. The positions of the permanent magnets are (±75, 0, 0) mm. (a) The helical robot is positioned at (0, 25, 0) mm. (b) The helical robot is positioned at (0, 0, 0) mm. (c) The helical robot is positioned at (0, −25, 0) mm. (d) The actuating magnetic field is subtracted from the total magnetic field to provide the robot's field at position (0, 25, 0) mm, (0, 0, 0) mm, and (0, −25, 0) mm, respectively. The red dot indicates the position of the helical robot between the rotating dipole fields. (e) Magnetic field is calculated vs the distance for permanent NdFeB cylindrical magnets with diameter and length *D*. The horizontal solid and dashed lines represent the theoretical resolution of 2 Hall-effect sensors with a resolution of 0.1 mT and 0.16 μT, respectively.

Figures 4(a)–4(c) show the representative simulation results of the actuating magnetic fields of the two rotating permanent magnets and the magnetic field of the robot when it is located at (0, 25, 0) mm, (0, 0, 0) mm, and (0, −25,0) mm. The magnetic field is calculated by superimposing (2) and (3), using the parameters provided in Table I. The fields are calculated at the plane of the Hall-effect sensors (*z *=* *3 mm) and for zero angular position of the rotating permanent magnets. This simulation indicates that the resultant magnetic field is approximately 5 mT at the position of the sensor (sensitivity is 0.1 mT). Figure 4(d) shows the magnetic field of the robot at *z *=* *3 mm for the three mentioned positions after subtraction of the actuating magnetic field. The magnetic field at the position of the sensor is one order of magnitude greater than its sensitivity. Figure 4(e) shows the relation between the size of the magnetic head of the helical robot and the ability of the sensor to measure its magnetic field.

To calculate the position of the helical robot using (2), we calculate the magnetic fields **B**_d1_ and **B**_d2_ using (3) and measure the magnetic field $B_{s}^{i}$ at the *i*th sensor. The position vector ($p_{s}^{i}$) to the *i*th sensor from a frame of reference is fixed. Therefore, the position vector of the helical robot is calculated using

$$p_{r}=p_{s}^{i}-p_{s-r}^{i},$$ (4)

where **p**_r_ is the position vector to the helical robot from a frame of reference, as shown in Fig. 3. In (4), $p_{s-r}^{i}$ is solved such that the following objective function is minimized:

$$\begin{array}{cc} \underset{p_{s-r}^{i}}{\text{minimize}} & \;\varepsilon ={\left({\hat{B}}_{r}-B_{r}\right)}^{T}({\hat{B}}_{r}-B_{r})\\ \;\text{subject}\;\text{to} & \;x^{2}+y^{2}-r^{2}=0, \end{array}$$ (5)

where ${\hat{B}}_{r}$ is the calculated magnetic field using (2) and **B**_r_ is determined using (1) based on the magnetic field measurement and the calculated actuating magnetic fields using (3). Further, *x* and *y* are the components of $p_{s-r}^{i}$, and *r* is the radius of the catheter segment (or rabbit aorta) that contains the helical robot. The radius of the catheter is included in the constraint equation to restrict the optimization search. This optimization routine is solved iteratively using the interior-method for constrained nonlinear optimization using C++, and a 15-point moving average filter is used for smoothing the estimated position. The filtered position is provided to a closed-loop motion control system.

### C. Closed-loop motion control of the helical robot

Two DC motors are used to rotate the permanent magnets, and the helical robot is allowed to rotate and swim at the center of the distance between the permanent magnets. The dynamics of these motors is given by

$$\frac{d}{dt}\left(\begin{array}{c} \omega_{k}\\ I_{k} \end{array}\right)=\left(\begin{array}{cc} -\frac{b}{J} & \frac{k}{J}\\ -\frac{k}{L} & -\frac{R}{L} \end{array}\right)\left(\begin{array}{c} \omega_{k}\\ I_{k} \end{array}\right)+\left(\begin{array}{c} 0\\ \frac{1}{L} \end{array}\right)u_{k}\;\text{for}\;k=1,2,$$ (6)

where *ω_k_* and *I_k_* are the angular velocity and input current of the *k*th DC motor, respectively. Further, *b*, *J*, and *k* are the motor viscous friction constant, moment of inertia of the rotating dipole field and the rotor of the motor, and torque constant, respectively. *L* and *R* are the electric inductance and resistance of the motor, respectively. The following control input is applied to synchronize the two rotating dipole fields:

$$u_{1}=k_{1}\left(\theta_{1}-\theta_{2}\right)+k_{2}\left(\omega_{1}-\omega_{2}\right),$$ (7)

where *k*_1_ and *k*_2_ are the proportional and derivative positive gains, respectively, and *θ_k_* is the angular position of the *k*th motor. Finally, the helical robot is controlled using the following control input:

$$u_{2}=k_{3}\left(\left\|p_{c}\right\|-\left\|p_{r}\right\|\right)+k_{4}\left(\left\|{\dot{p}}_{c}\right\|-\left\|{\dot{p}}_{r}\right\|\right),$$ (8)

where *k*_3_ and *k*_4_ are positive proportional and derivative gains, and **p**_c_ is the position of the blood clot. In (8), **p**_r_ is estimated and used in the closed-loop motion control.

## II. CONTROL AND REMOVAL OF BLOOD CLOTS

In order to examine the validation of the magnetic tracking, the helical robot is allowed to swim inside *in vitro* and *ex vivo* models and magnetic localization is implemented.

### A. Localization and motion control *in vitro*

**FIG. 5.**
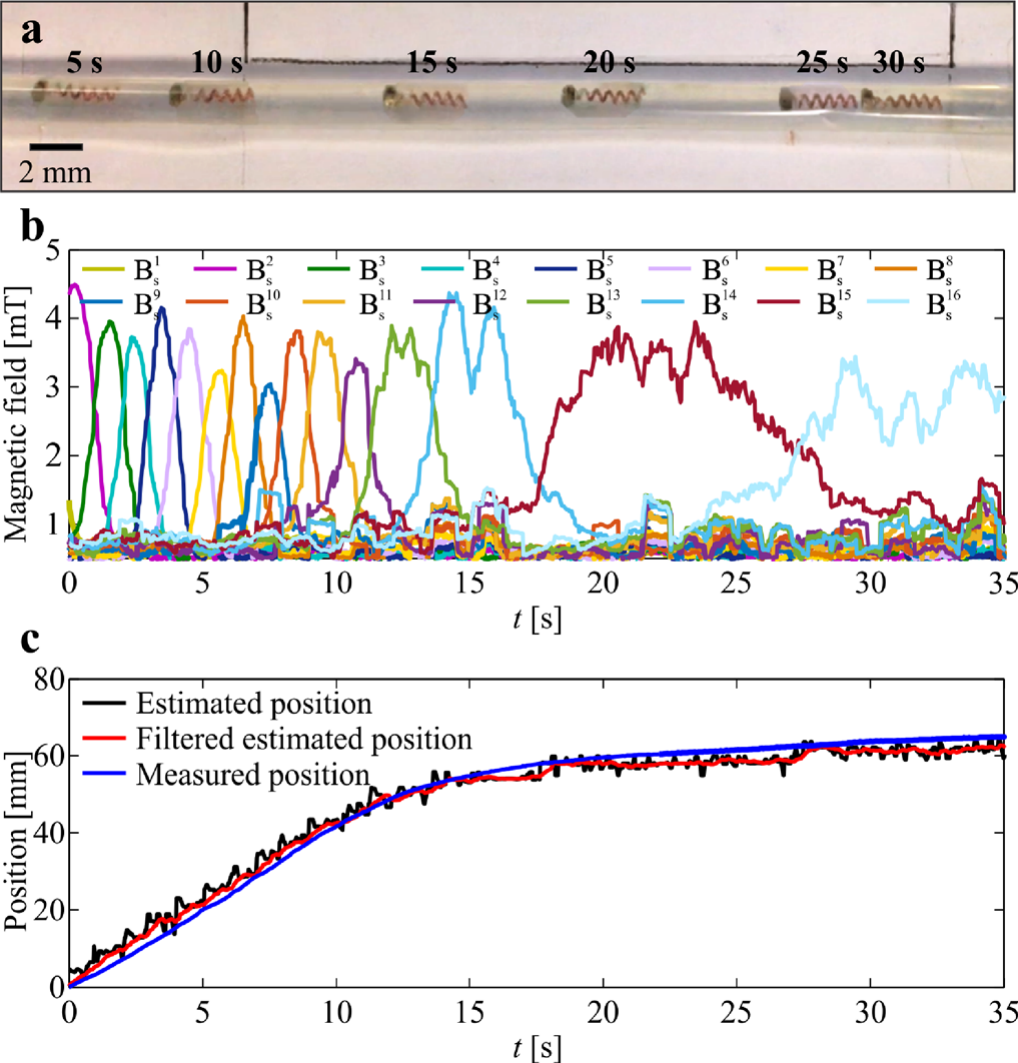
Position of a helical robot is tracked continuously during propulsion along a catheter segment. (a) The helical robot swims at an average speed of 4.2 mm/s under the influence of a rotating magnetic field at a frequency of 5 Hz. (b) Magnetic field is measured using an array of 16 Hall-effect sensors. $B_{s}^{i}$ is the magnitude of the three magnetic field components measured at the *i*th sensor. (c) The estimated position (filtered using 15-point moving average filter) of the helical robot is compared to the measured position using computer vision. The absolute position error is 2.32 mm. (See the supplementary material video).

**FIG. 6.**
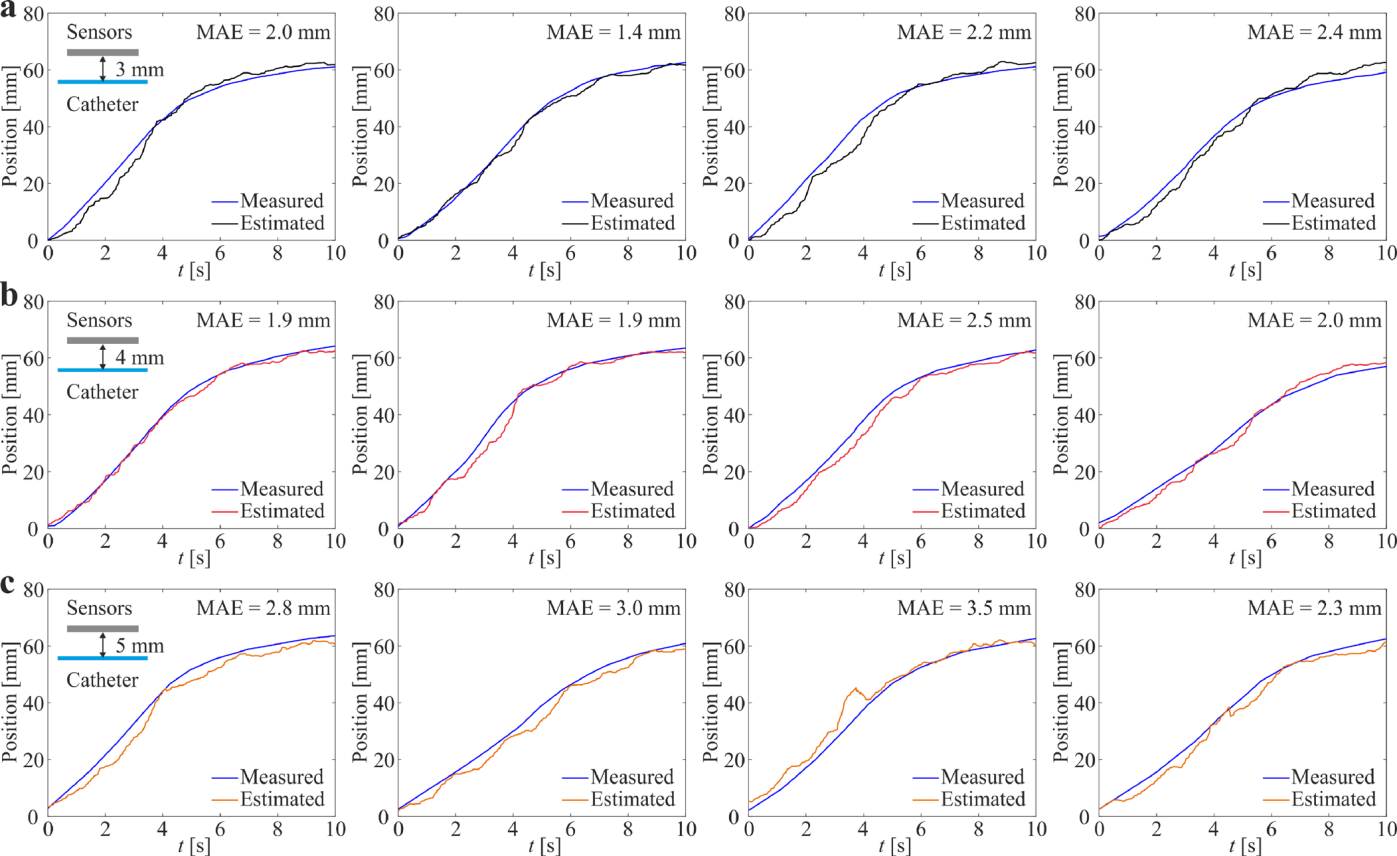
Magnetic localization of the helical robot is achieved at different distances between the center of the catheter segment and the array of Hall-effect sensors. Position of the helical robot is measured by visual feedback (blue line) and compared to the result of the magnetic localization using Eqs. (4) and (5). The robot swims under the influence of rotating fields at an actuation frequency of 5 Hz. (a) For distance of 3 mm, the mean absolute error (MAE) is measured as 1.8 ± 0.5 mm (*n *=* *5). (b) For distance of 4 mm, the MAE = 2.2 ± 0.4 (*n *=* *5). (c) For distance of 5 mm, the MAE = 3.0 ± 0.5 (*n *=* *5).

The helical robot is allowed to swim inside a catheter segment under the influence of a rotating magnetic field at frequency of 5 Hz, as shown in Fig. 5(a). The measured magnetic field using the Hall-effect sensors and the precalculated magnetic field map are used in the objective function (5) to calculate $p_{s-r}^{i}$. The magnetic field measurements during the movement of the helical robot are shown in Fig. 5(b). Each Hall-effect sensor provides a maximum magnetic field measurement when the robot is close to its tip. The maximum magnetic field is measured as 4.5 mT, whereas the minimum field measured by two adjacent sensors is 1.6 mT during the movement of the robot with respect to the sensors. Even though the catheter segment is aligned with the linear array of the Hall-effect sensors, we observe that the peak provided by each sensor is different owing to the nonuniform swimming speed of the helical robot along the catheter. In these trials, the helical robot is actuated using rotating magnetic fields at a frequency of 5 Hz. Nevertheless, there exists a nonuniform magnetic force along the propulsion axis as shown in Figs. 4(a)–4(c). This force contributes to the time-varying speed of the robot for the same actuation frequency and the deviation between the measured peaks between the adjacent sensors. Position of the helical robot is tracked using visual feedback and feature tracking^34^ to validate the magnetic localization, as shown in Fig. 5(c). In this representative experiment, the mean absolute error (MAE) is 2.32 mm. This experiment is repeated 20 times, and the absolute position error is calculated as 2.35 ± 0.4 mm. The position of the helical robot is estimated using three representative distances between the center of the catheter segment and the linear array of Hall-effect sensors, as shown in Fig. 6. We observe that the MAE of the magnetic localization increases with the distance between the sensor array and catheter. For a distance of 3 mm, the MAE is measured as 1.8 ± 0.5 mm (*n *=* *5), as shown in Fig. 6(a). The measured MAE increases to 2.2 ± 0.4 (*n *=* *5) for a distance of 4 mm owing to the decrease in signal-to-noise (SNR) with the distance [Fig. 6(b)]. At distance of 5 mm, the MAE is measured as 3.0 ± 0.5 (*n *=* *5), as shown in Fig. 6(c).

**FIG. 7.**
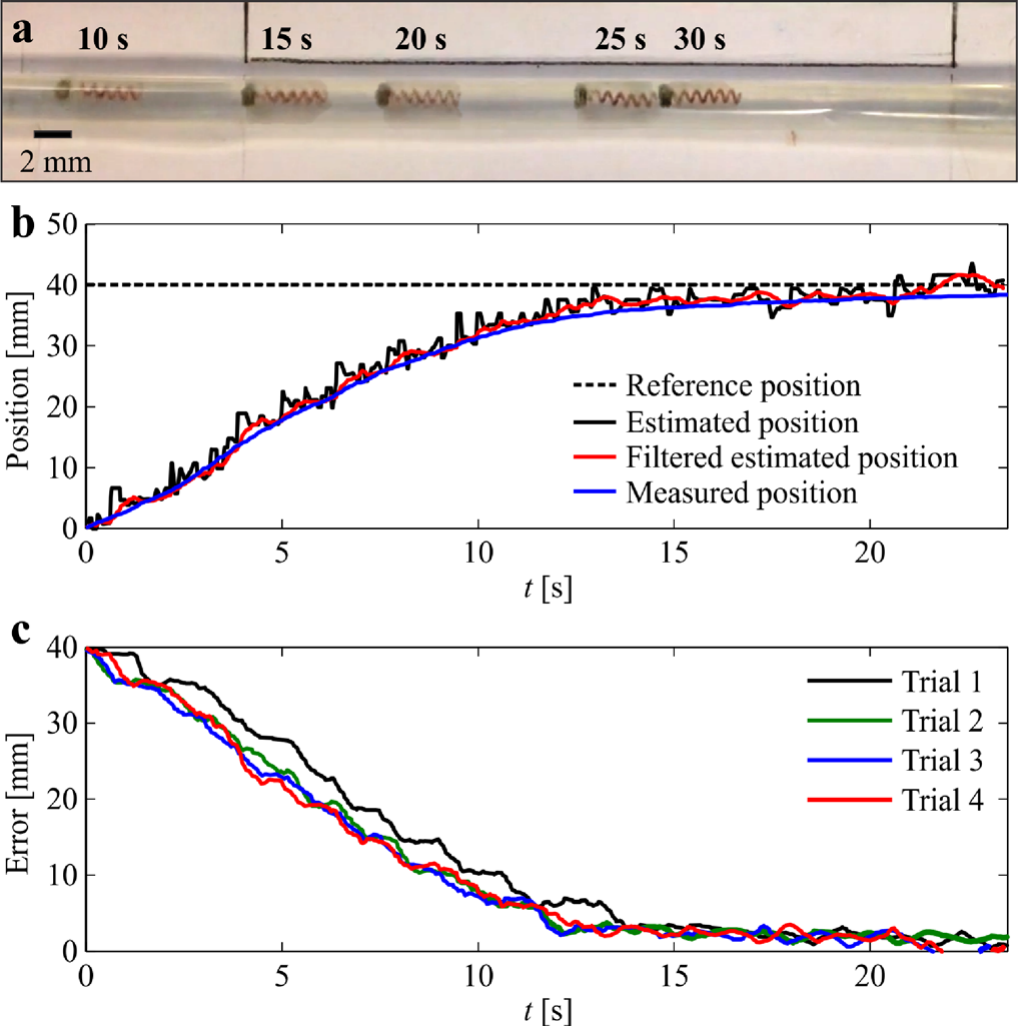
Closed-loop motion control of a helical robot is achieved inside *in vitro* model. (a) The helical robot swims toward a reference position inside a catheter segment. (b) The estimated position (filtered using 15-point moving average filter) of the helical robot is used in the control law (8). (c) The average position error is 0.74 ± 1.9 mm (*n *=* *10) (see the supplementary material video).

This localization error is due to the signal-to-noise (SNR) ratio. The SNR decreases as the distance between the helical robot and Hall-effect sensor increases. In addition, deviations between the applied magnetic field and the precalculated magnetic field map also contribute to the localization error of the helical robot. To determine the deviation between the precalculated magnetic field map and applied magnetic field, we measure the magnetic field using the 16 Hall-effect sensor in the absence of helical robots. The measured magnetic field is subtracted from the precalculated magnetic field map. The average error between the measured magnetic field and the precalculated magnetic fields is 0.67 ± 0.09 mT. Therefore, the localization performance can be improved with accurate field modeling and higher SNR (via magnetic sensors with higher sensitivity). The estimated position of the helical robot is used in (8) to achieve closed-loop motion control, as shown in Fig. 7. In this experiment, the estimated position and reference positions are provided to control law (8), and control law (7) is implemented to synchronize the two rotating dipole fields. Figure 7(a) shows the response of the helical robot at different time instants. The estimated and measured positions are provided in Fig. 7(b). The helical robot is positioned at the reference (dashed black line) with an average steady-state error of 0.74 ± 1.9 mm (*n *=* *10). This closed-loop control procedure is followed by mechanical rubbing of the blood clot (see supplementary material video).

### B. Clearing of blood clots

**FIG. 8.**
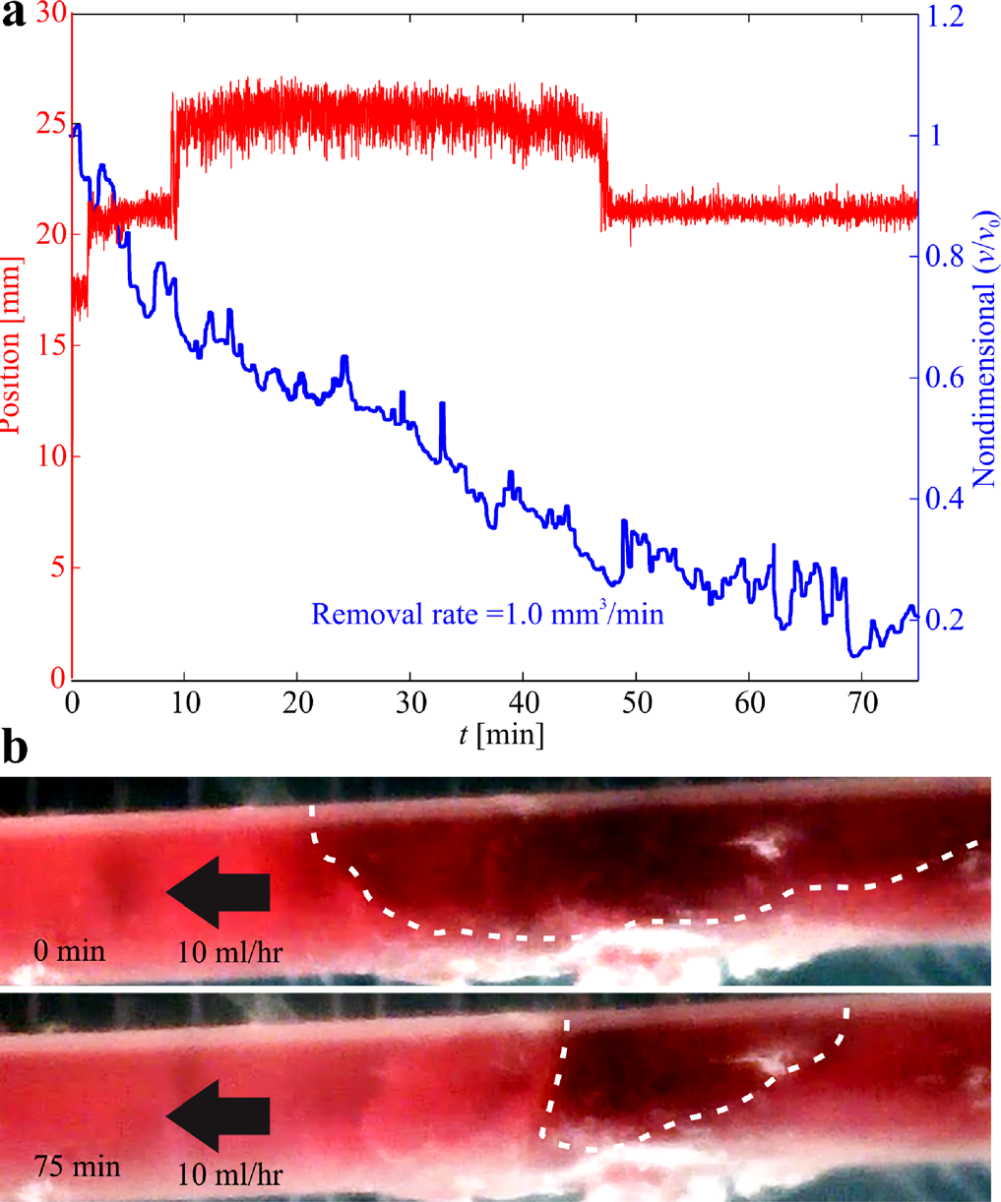
1-h-old blood clot is mechanically removed using a helical robot. The robot is controlled toward the clot and mechanical removal is achieved at an actuation frequency of 5 Hz, and against flow rate of 10 ml/h. (a) Position of the helical robot is estimated using magnetic tracking and used in the motion control system. The initial volume of the clot (*v*_0_) is 94.24 mm^3^. The size of the blood clot (*v*) is decreased by 60.8% and 79.7% following 40 min and 75 min of mechanical rubbing, respectively. (b) The dashed white lines indicate the pre-conditions and post-conditions of the blood clot (see the supplementary material video).

1-h-old blood clot samples are prepared (preparation protocol is approved by the local Institutional Review Board) and inserted inside the catheter segment.^26^ The initial volume (*v*_0_) of the clot is 94.24 mm^3^ (length and diameter of 7.5 mm and 4 mm, respectively) and the volume is measured throughout each trial via visual feedback.^26^ Figure 8 shows a representative experimental result of clearing a clot under the influence of a rotating magnetic field at a frequency of 5 Hz. The position of the helical robot is estimated using our magnetic tracking method. Although this experiment is done inside a catheter segment, visibility of the helical robot is relatively low due to the dissolution of the blood clot by the helical robot, as shown in Fig. 8. Nevertheless, the magnetic-based localization provides an estimate of the position of the helical robot along the catheter throughout the clearing procedure of the clot. The closed-loop control achieves a rise time of 7 s (time to reach the blood clot). Once the helical robot comes into contact with the clot, it does not move forward and its tip tears the fibrin network of the clot. After approximately 1.5 min, the helical robot penetrates the clot with a depth of 3 mm. We observe a similar behavior at time, *t *=* *10 min. At time *t *=* *47 min, the clot is cleared and the robot is pushed back by the flowing streams of the PBS. The size of the blood clot is decreased by 60.8% and 79.7% after 40 min and 75 min of mechanical rubbing, respectively (see supplementary material video).

### C. Localization and motion control *ex vivo*

**FIG. 9.**
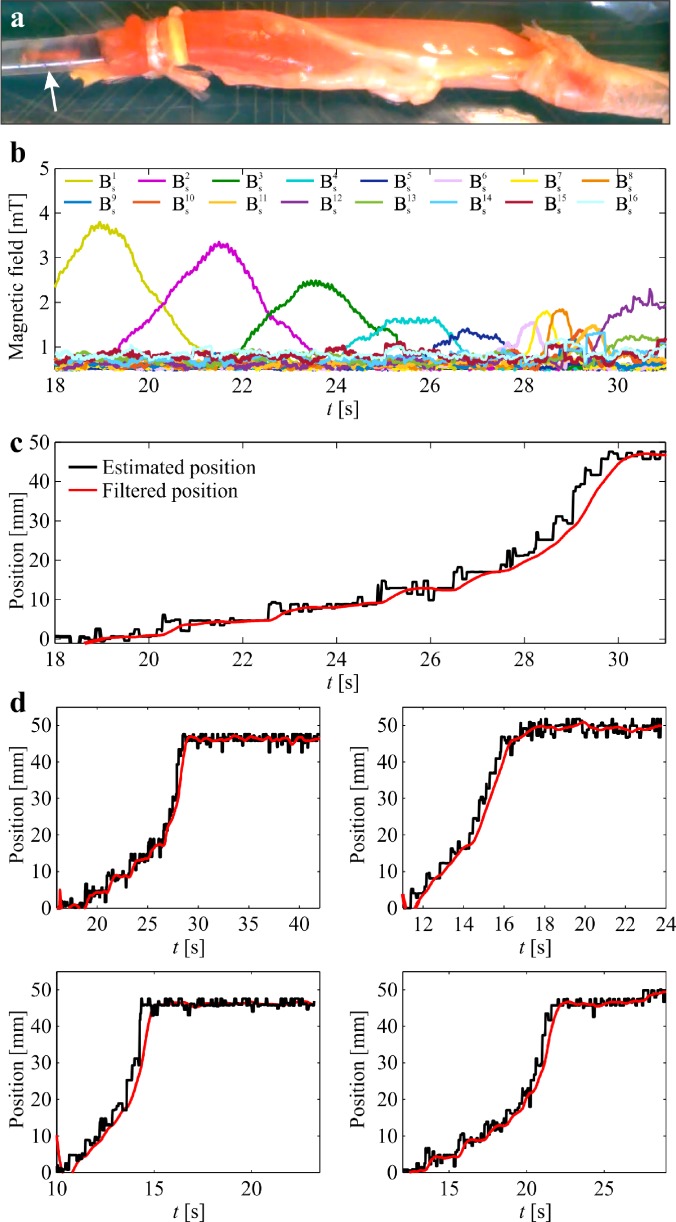
Position of a helical robot is tracked continuously during propulsion inside a rabbit aorta. (a) The helical robot swims at an average speed of 4.4 mm/s under the influence of a rotating magnetic field at frequency of 5 Hz. (b) Magnetic field is measured using an array of 16 Hall-effect sensors. $B_{s}^{i}$ is the magnitude of the three magnetic field components measured at the *i*th sensor. (c) The estimated position of the helical robot is compared to the calculated position using ultrasound feedback. A 15-point moving average filter is used for smoothing the data. (d) The average speed of the helical robot inside the rabbit aorta is 7.1 ± 3.4 mm/s (see the supplementary material video).

In order to characterize the magnetic localization inside a real blood vessel, a segment from the aorta is isolated from a rabbit and connected to the catheter [Fig. 9(a)] to provide a flow rate of 10 ml/h. Aorta is the main artery that originates in the heart and delivers oxygenated blood to the organs. The diameter of rabbit aorta fits the catheter we are using to deliver the flow, and the use of arteries is clinically relevant since the major cause of ischemic diseases such as stroke and myocardial infarction is the obstruction of the corresponding artery by blood clots. Figure 9(b) shows the measured magnetic fields of the Hall-effect sensor array during a representative open-loop trial under the influence of a rotating magnetic field at frequency of 5 Hz. The corresponding estimated position of the helical robot inside the aorta is shown in Fig. 9(c). In this representative trial, the average speed of the helical robot is 4.4 mm/s against the flow rate of 10 ml/h. This experiment is repeated [Fig. 9(d)] inside the aorta and the average speed of the helical robot is measured as 7.1 ± 3.4 mm/s (*n *=* *5).

**FIG. 10.**
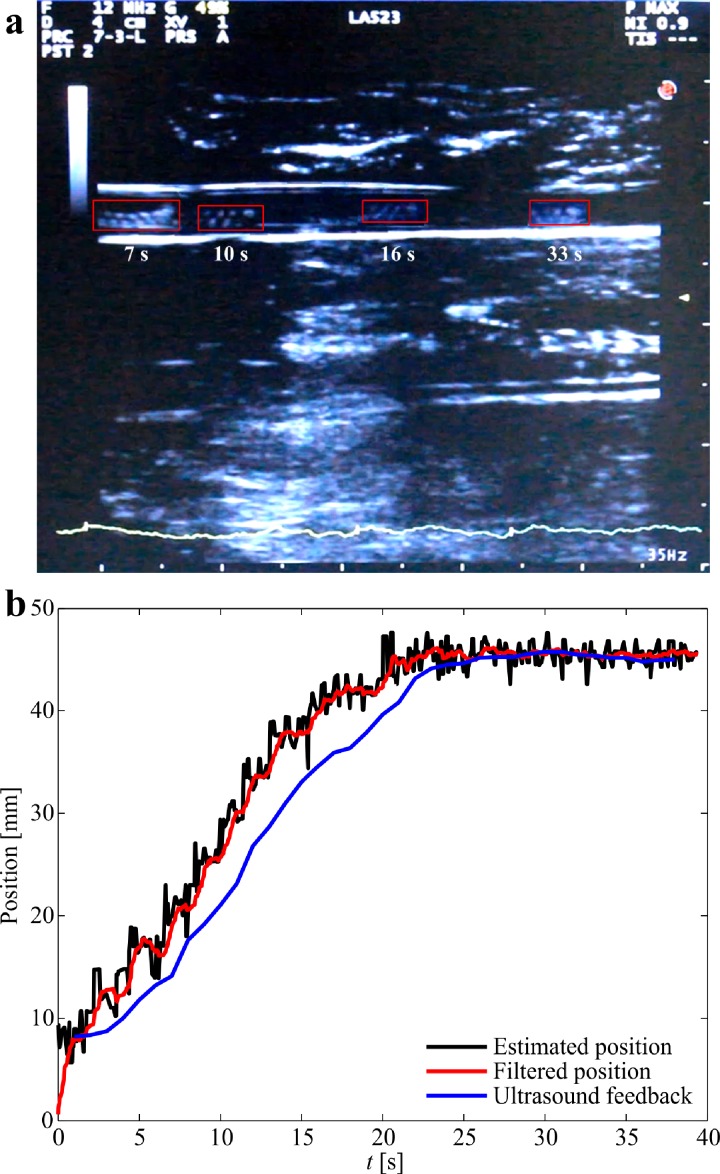
Magnetic localization of a helical robot is achieved and compared to ultrasound feedback. (a) The helical robot is allowed to swim in whole blood and its position is detected using an ultrasound transducer (LA523 linear array ultrasound transducer, Esaote, Italy). The red rectangles indicate the position of the helical robot at different time instants. (b) The absolute position error between the magnetic tracking and ultrasound feedback is 2.6 mm (see the supplementary material video).

In order to validate the accuracy of the magnetic tracking during *ex vivo* trials, an ultrasound transducer is incorporated to localize the helical robot. The catheter segment (or the rabbit aorta) is filled with whole blood and contained in a gelatin reservoir to achieve air-free coupling with the transducer, as shown in Fig. 2(c). The reservoir is fixed above the Hall-effect sensor array. The position of the helical robot is localized simultaneously using ultrasound feedback and magnetic tracking. Figure 10(a) shows the motion of the helical robot using ultrasound feedback for a depth of 5 cm. The frequency of the ultrasound waves is set to 12 MHz, and the ultrasound system is adjusted to motion mode (*M-mode*) to acquire scans during propulsion. The thermal index score (TIS), mechanical index (MI), and gain are 0.1%, 0.9%, and 49%, respectively. The position of the helical robot is tracked from the acquired ultrasound scans and compared to the estimated position of the magnetic localization, as shown Fig. 10(b). The absolute position error between the ultrasound and magnetic localization is 2.6 mm. This error is approximately equal to the error between the measured position using visual feedback and magnetic tracking. Again, this error can be attributed to the field modeling errors and the sensor background noise (see supplementary material video).

It is expected that the flow past the helical robot and the blood clot increases as the volume of the clot decreases. Therefore, the propulsive force of the helical robot and the magnetic force of the actuation system must hold the robot against the flowing streams of the fluid. At an actuation frequency of 5 Hz, the helical robot achieves zero net displacement against a flow rate of 800 ml/h. The helical robot can overcome greater flow rates by increasing the actuation frequency. At actuation frequency in the range of 6 Hz to 8 Hz, the propulsive force enables the robot to overcome the flow rate of approximately 900 ml/h.

## III. DISCUSSIONS

Superficial veins of the legs are located in the subcutaneous tissue beneath the skin with a variable thickness among individuals. Their depth is estimated to vary from 1.48 mm to 31.6 mm in the region of the anterior thigh in adults,^29^ where the great saphenous vein circulates. SVT should not be underestimated due to the risk of propagation into the deep veins of the leg, resulting in deep vein thrombosis (DVT) with risk of fatal lung complications.^27^ We have demonstrated magnetic localization and closed-loop motion control of the helical robot inside *in vitro* and *ex vivo* models of blood clots. However, the localization and control of robots *in vivo* remains a challenge. In this work, an array of Hall-effect sensors is used to localize a helical robot with a diameter of 1 mm as a noninvasive magnetic localization method. The localization accuracy is validated using visual and ultrasound feedback for the *in vitro* and *ex vivo* conditions, respectively. Although the depth range detected by the sensors is limited to 5 mm, our results support the feasibility to localize and control robots *in vivo* for the mechanical removal of blood clots in the superficial veins of the leg. Several challenges have to be overcome to target realistic clinical conditions such as DVT of the legs or arterial thrombosis. First, the workspace is currently limited by the relatively low SNR as the distance between the sensor and helical robot increases. Therefore, magnetic field sensors with a greater range have to be tested and the position of the robot has to be estimated through an optimal filter.^22^ Second, magnetic localization is implemented using a linear array of Hall-effect sensors, that is, difficult to align with blood vessels in real *in vivo* applications. Therefore, it is essential to use a planar or three-dimensional array of sensors to enhance the localization of the helical robot during propulsion inside real vessels with bifurcations. Third, our experimental results are conduced against the flow rate of 10 ml/h. This flow rate is greater than blood flow in small arteriole, capillaries, and venule only. Therefore, it is essential to modify our permanent magnet-based robotic system to enable mechanical removal of blood clots against greater flow rates comparable to medium arteries and veins.

The experimental results in Fig. 10 reveal a fundamental difference between magnetic and ultrasound-based localization. The major limitations of ultrasound-based localization depend on its low SNR due to bony structures and air pockets within the tissue, or any other ultrasound wave reflectors. Magnetic fields are transparent to these wave reflectors and its SNR is only related to the size of the magnetic head of the helical robot in magnetic localization. In addition, the size of the helical robot does not represent a limitation in magnetic localization due to the availability of magnetic sensors in the range of microteslas to nanoteslas. The measured MAE in our experiment increases with the distance between the Hall-effect sensors and the helical robot (Fig. 6) due to its limited resolution (0.1 mT). In ultrasound localization, adequate resolution can only be achieved at relatively high frequencies of the propagating ultrasound waves, which is inversely proportional to the wavelength. Therefore, the size of the helical robot represents a limitation for these two minimally invasive localization techniques.

The localization and motion control of helical robots have been validated in the presence of a blood clot, mimicking the conditions of SVT. Not only do we observe that the size of the blood clot is significantly decreased (by 60.8% and 79.7% after 40 min and 75 min of mechanical rubbing, respectively), but we also acquire the position and observe the behavior (Fig. 8) of the helical robot throughout the clearing procedure of the clot by magnetic tracking, without relying on visual feedback. The magnetic tracking of the helical robot can also be used in its retrieval. Helical robots are likely to access the blood clot by insertion via a flexible catheter. Therefore, they can also be retrieved by swimming back controllably to the insertion point by magnetic localization.

## IV. CONCLUSIONS AND FUTURE WORK

In this paper, we implement a noninvasive magnetic localization method^24^ of a helical robot with a diameter of 1 mm for clearing superficial blood clots. The localization accuracy is characterized using visual feedback with a position tracking error of 2.35 ± 0.4 mm (*n *=* *20). Closed-loop motion control is achieved based on the estimated position of the robot toward blood clots *in vitro* with an average steady-state error of 0.74 ± 1.9 mm (*n *=* *10). Localization of the helical robot is also demonstrated inside a rabbit aorta and compared to the results of ultrasound feedback. The absolute position error between ultrasound and magnetic localization is 2.6 mm. The localization and control of the helical robot enables the removal of blood clots at an average removal rate of 0.67 ± 0.47 mm^3^/min.

As part of future studies, helical robots will be localized at a relatively large distance from the Hall-effect sensors. This modification is necessary to clear blood clots in deep veins [Fig. 4(e)] while still maintaining a closed-loop control of the helical robot. The distance between the sensor and the helical robot is currently limited owing to the lower SNR as this distance increases. Therefore, we will use magnetic field sensors with higher sensitivity to implement this approach on deep veins. We will also study the influence of rubbing in combination with chemical lysis at different doses of a fibrinolytic agent. The comparative study between mechanical rubbing, rubbing in combination with different percentages of fibrinolytic agent, and pure chemical lysis is essential to optimize the integration between mechanical rubbing and chemical lysis.

## V. METHODS

Local Institutional Ethical Board approval (2018–06-PBT-NH) of the Faculty of Pharmacy and Biotechnology is obtained for the preparation protocol of the blood clots, and donors gave written informed consent.

## SUPPLEMENTARY MATERIAL

See supplementary material video for the localization experiment (Fig. 5), closed-loop control experiment (Fig. 7), removal of the blood clot (Fig. 8), localization in the *ex vivo* model (Fig. 9), and localization using ultrasound feedback (Fig. 10).
